# Integrative fault diagnostic analytics in transformer windings: Leveraging logistic regression, discrete wavelet transform, and neural networks

**DOI:** 10.1016/j.heliyon.2025.e42872

**Published:** 2025-02-20

**Authors:** Salman Baroumand, Ali Reza Abbasi, Mohammadreza Mahmoudi

**Affiliations:** a Department of Electrical Engineering, Faculty of Engineering, Fasa University, Fasa, Iran; b Department of Statistics, Faculty of Science, Fasa University, Fasa, Iran

**Keywords:** Power transformer, Artificial neural network, Discrete wavelet transform, Logistic regression technique, Fault detection

## Abstract

Protection of transformers is crucial in the power industry due to their susceptibility to various electrical and mechanical faults over time. Traditional methods like Frequency Response Analysis (FRA) have limitations in accurately diagnosing these faults. This paper highlights the potential of combining advanced signal processing techniques with machine learning algorithms by presenting an innovative hybrid model for accurately detecting transformer winding faults, utilizing Logistic Regression, Artificial Neural Networks (ANN) and Discrete Wavelet Transform (DWT). The primary novelty of this approach lies in the use of Logistic Regression to evaluate the impact of each wavelet decomposition, which aids in selecting the most effective wavelet bases, reducing data volume, and decreasing computational complexity. By integrating these methods, the proposed model significantly enhances fault detection accuracy and system performance. The effectiveness of the algorithm is validated through a practical case study, demonstrating a 97 % success rate in detecting transformer faults and reducing misclassification to 2.9 %.

## 1 Introduction

In the complex power systems, transformers are critical yet vulnerable to disturbances that can cause mechanical and electrical damage, leading to significant economic and social impacts [1]. Faults in transformer windings, often resulting from factors like short circuits, mishandling, or explosions, can cause various structural damages [2]. Undetected, these faults can have dire consequences, including environmental harm and power outages. Thus, accurate and early fault detection, facilitated by advanced diagnostic tools and monitoring systems, is crucial to maintain system reliability and minimize downtime costs [3,4,5]. Recent research has produced a variety of diagnostic methods, each designed to meet specific challenges in detecting transformer faults, highlighting the importance of early fault detection to prevent transformer failures. Furthermore, a significant body of research has been dedicated to the advancement and refinement of diagnostic methodologies. Techniques such as Transfer Function (TF) analysis and Frequency Response Analysis (FRA) have been extensively employed to ascertain the presence of electrical and mechanical anomalies in transformers [6,7,8,9]. The TF approach, a method reliant on comparative analysis, utilizes four principal algorithms. These algorithms are predominantly grounded in estimation methods, artificial exact calculations, electric circuit models and intelligent algorithms [10,11,12]. The TF method assesses new data by comparing it with a reference point. Significant variances in this comparison suggest a malfunction within the transformer, necessitating thorough checks to identify the fault's nature, location, and intensity. In Ref. [13], the authors have introduced three standard approaches to acquire the TF for transformer winding evaluation. These include comparisons of TF profiles based on type, structure, and timing, all of which require manual analysis by experienced specialists. This technique presents certain limitations, notably its inability to identify minor and initial alterations in winding distortions, and it requires the expertise of a specialist for result interpretation. Additionally, dissolved gas analysis (DGA) is another methodology extensively referenced in scholarly works for the detection of internal defects in power transformers [14,15,16].

While online DGA monitoring exists, it requires further improvement for more effective real-time monitoring and fault diagnosis. On the other hands, traditional DGA interpretation methods like the Duval triangle may not include all relevant gases such as ethane and hydrogen, which are important for diagnosing certain fault types and Decision-Making Limitations. Moreover, some DGA techniques, including Doernenburg and Roger's ratios, as well as IEC standards, may lead to ‘no decision’ scenarios for cases that fall outside of specified codes [17]. So, accurate DGA analysis is still a gap in the field of condition monitoring of power transformer. To address fault classification and related challenges, researchers have employed a spectrum of artificial intelligence methods. Studies in Refs. [18,19] have explored the concurrent use of intelligent and analytical techniques to detect faults through the analysis of current and voltage signals. A variety of intelligent approaches, including fuzzy logic classifiers [18], artificial neural networks (ANN) [19,20], and Bayesian classifiers [21], have been introduced for fault identification and differentiation. Transformations such as time-time [22], Chirplet [23], S-transform [24], support vector machine [25], and wavelet transforms like Discrete Wavelet Transform (DWT) and MODWT [26,27,28] are prevalent for feature extraction in training classifiers. The Wavelet Transform (WT) [29,30], known for extracting detailed signal information, has gained traction for classification, pattern recognition, and signal processing tasks. The Wavelet Neural Network (WNN) [31,32] merges neural networks' learning abilities with WT's precision, enhancing its nonlinear function approximation, crucial for estimations and predictive analytics. The ‘waveNet’ [33,34] and ‘wavelet network’ [31] methods process neural networks and wavelets, with the former analyzing signals via DWT and the latter optimizing neural network parameters. Additionally, principal component analysis has been leveraged for transformer protection and diagnosing power quality issues in distribution systems [35,36,37,38,39,40,41,42]. One of the main challenges in using DWT in these methods is selecting the appropriate wavelet bases for signal decomposition. Incorrect selection can lead to reduced fault detection accuracy. Logistic Regression can serve as a tool to help select the most effective wavelet bases, reduce data volume, and decrease computational complexity. In other words, it optimally prepares the data for integration with DWT and ANN, thereby enhancing the accuracy and speed of fault detection.

In this study, an innovative method for detecting transformer winding faults is introduced by integrating logistic regression, discrete wavelet transform (DWT), and artificial neural networks (ANN). The discrete wavelet transform (DWT) is applied to the frequency response data to extract relevant features. Logistic regression is then used to evaluate the impact of each wavelet decomposition. The goal is to identify the most effective wavelet bases and reduce data volume to improve computational efficiency and fault detection accuracy. The features extracted from the DWT are used as inputs to an artificial neural network (ANN). This network is trained using healthy and faulty data to develop a diagnostic model capable of accurately identifying and classifying transformer faults. The performance of the ANN is evaluated using confusion matrices and other statistical metrics, including accuracy, sensitivity, and specificity. On the other hand, this research employs several methods to manage and reduce noise in the input signals: using a swept frequency input signal to improve the signal-to-noise ratio (SNR), applying Discrete Wavelet Transform (DWT) to decompose signals and eliminate frequency noise, evaluating wavelet decompositions with logistic regression to select the most effective wavelet bases, and normalizing input data for the artificial neural network (ANN) to enhance fault detection accuracy. These combined approaches ensure cleaner input signals and improved system performance and their goal is to ensure the accuracy and reliability of the diagnostic model in identifying and classifying transformer faults.

The key innovations presented in this research are as follows:

• By integrating Logistic Regression, DWT, and ANN, the accuracy and reliability in fault diagnosis are significantly enhanced. This optimized hybrid method provides better performance compared to existing methods and yields more precise results in transformer fault diagnosis.
• One of the main innovations of this article is the use of Logistic Regression to evaluate the impact of each wavelet decomposition in the classification method. This approach allows us to identify and select the most impactful wavelet bases, which helps improve the accuracy and efficiency of the fault diagnosis system.
• By using the selected wavelet bases that have a greater impact, the volume of input data to the system is significantly reduced. This data reduction not only simplifies the data processing but also optimizes the overall performance of the system.
• The selection of more impactful wavelet bases and the reduction of input data lead to a decrease in computational load and the size of the neural network. This reduction in computational complexity increases the training speed of the neural network and improves the system's efficiency.
• The combination of Logistic Regression with DWT and ANN is introduced as a new approach in this article. This methodological innovation presents a unique hybrid method that has been less explored in the scientific literature and leads to significant advancements in the field of transformer fault diagnosis.

This research is organized as follows: Section 2 delves into the power transformer model and transfer function, elaborating on the proposed methodology. Section 3 lays out the foundational aspects of the employed technique and explicates its application. Section 4 is dedicated to the exposition and simulation results of a case study involving a 10/0.4 kV, 1.2MVA transformer. Following this, numerical simulations are executed to assess the method, utilizing both a benchmark classification dataset and datasets related to power transformer faults. The paper concludes with the final section.

## 2 Problem description

Diverse strategies are applied to track the condition of power transformers. Among these, Frequency Response Analysis (FRA) is notably effective for the precise tracking of issues like short circuit turns, as well as the displacement and deformation of windings. Through the examination of the transformer's transfer function, one can derive frequency response curves that reflect both the present and reference conditions. The fundamental components of the transformer—namely the core, windings, and insulation—are represented by corresponding electrical parameters, including inductances, capacitances, and resistances. Should there be any mechanical or electrical disturbances within the winding, these electrical parameters would undergo modifications, thereby affecting the frequency response of the winding. Even a minor change in the configuration of the windings can affect the electrical parameters, thereby influencing the transfer function of the windings.

### 2.1 Transfer function

**Fig. 1:**
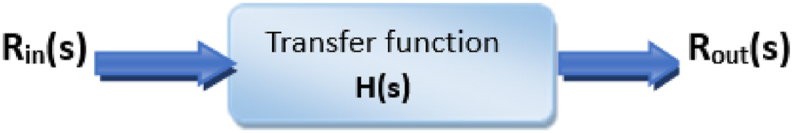
System representation of a transfer function (TF).

Dick and Erven [43] suggested the transfer function (TF), a widely-adopted comparative technique. This method evaluates new data against a standard to identify and analyze any significant variances. It's based on the “fingerprint” concept, which asserts that each transformer's TF is unique. A transformer's individual frequency response, determined by its inductive and capacitive elements, is unique to its construction. The TF waveforms vary with the windings' architecture, and each type of winding has a typical TF signature. Resonance peaks at certain frequencies, due to the interaction of leakage inductance and capacitance, can pinpoint transformer anomalies. In mathematical terms, the TF is the ratio of system output to input when initial conditions are zero, in a time-invariant linear system. The system's transfer function representation is depicted in Fig. 1, where H(s), R_in_(s), and R_out_(s) correspond to the system's transfer function within the Laplace transform domain, as well as the input and output signals, respectively.

TF can be mathematically stated as follows:

(1) $$H\left(s\right)=\frac{R_{out}\left(s\right)}{R_{in}\left(s\right)}$$

The transfer function (Eq. (1)) is inherently linked to the system's parameters, independent of the signals received or produced. It reveals how the magnitude and phase angle fluctuate with frequency. Mechanical issues like axial and radial shifts or winding short circuits can alter the TF by changing the resonance frequency's intensity and position. These alterations cause a shift in the TF's poles and zeros. The TF is crucial for examining winding deformations in transformers, as it reflects the relationship between changes in electrical parameters and physical distortions of the windings. Consequently, mechanical impairments in the winding's structure are detectable by analyzing variations in the TFs.

The TF approach is applicable within both time and frequency domains. Typically, the identification of faults is performed by examining the rational functions within the frequency domain. The key Transfer Functions are enumerated below:

i. *Admittance transfer function:* It is obtained by taking the ratio of the input current (I_input_) to the input voltage (V_input_) when both measurements are referring to the same winding of the transformer. This can be mathematically represented as Eq. (2):

(2) $$TF\left(f\right)=\frac{I_{\text{input}}\left(f\right)}{U_{\text{input}}\left(f\right)}$$

ii. *Voltage or current transfer function:* By dividing the output voltage into the input voltage or the output current into the input voltage, we get the TFs for voltage (Eq. (3)) or current (Eq. (4)) respectively as bellow*:*

(3) $$TF\left(f\right)=\frac{U_{\text{output}}\left(f\right)}{U_{\text{input}}\left(f\right)}$$

(4) $$TF\left(f\right)=\frac{I_{\text{output}}\left(f\right)}{U_{\text{input}}\left(f\right)}$$

Here U_input_, U_output_, *f,* I_input_ and I_output_ are the supply voltage, output voltage, frequency, transformer input and output currents; respectively. These relationships are crucial for assessing the electrical integrity of transformer windings and for identifying any anomalies that may suggest the presence of a fault. In practice, applying a specified voltage to a transformer's winding and observing the corresponding current allows for the computation of the admittance transfer function. Any deviation from the predicted values could indicate a defect in the winding, potentially due to a short circuit or deformation. The primary obstacle with TF is that their comparison is visual, necessitating expert analysis, and the quality of the results is contingent upon the expert's experience and proficiency, as noted in Ref. [7].

## 3 Background of methods employed

### 3.1 Wavelet decomposition

In the realm of frequency domain analysis, pinpointing low-frequency elements poses a challenge, whereas discerning high-frequency elements is more arduous in the time domain. Wavelet Transforms (WT) adeptly address these challenges. The principal feature of the DWT, a variant of WT, is its ability to balance frequency and time resolution. DWT stands as a versatile tool in signal processing, widely employed across diverse engineering fields, including power systems [24,36,33,38]. DWTs are advantageous because they provide greater frequency resolution at lower frequencies and improved time resolution at higher frequencies. This is achieved by decomposing the signal into an approximation and detailed components. Eq. (5) delineates the DWT for the discrete signal (*x*(k)):

(5) $$\text{DWT}\left(x\left(k\right),m,n\right)=\left(a_{h}\left|d_{h}\right|d_{h‐1}\left|\ldots \left|d_{3}\right|d_{2}\right|d_{1}\right)=\frac{1}{\sqrt{2^{m}}}\sum_{k}x\left(k\right)\psi \left(\frac{n‐k2^{m}}{2^{m}}\right)$$

where the mother wavelet is ψ(.), and m and n are the parameters for the time scale, *h* is number of decomposition Level (or number of component details) and 2^m^ and k2^m^ are the variable for scaling and shifting. Also $a_{h}$ and $d_{1}$… $d_{h}$ are signal approximation and detail components. So at the last decomposition level *h*, the original signal *x*(*k*) can be rebuilt by making and adding up all the rough and fine parts signals as Eq. (6):

(6) $$x\left(k\right)=c_{1}d_{1}\left(k\right)+c_{1}a_{1}\left(k\right)=c_{1}d_{1}\left(k\right)+c_{2}d_{2}\left(k\right)+c_{2}a_{2}\left(k\right)=\ldots =c_{1}d_{1}\left(k\right)+c_{2}d_{2}\left(k\right)+c_{3}d_{3}\left(k\right)+\ldots +c_{h}d_{h}\left(k\right)+c_{h}a_{h}\left(k\right)$$

**Fig. 2:**
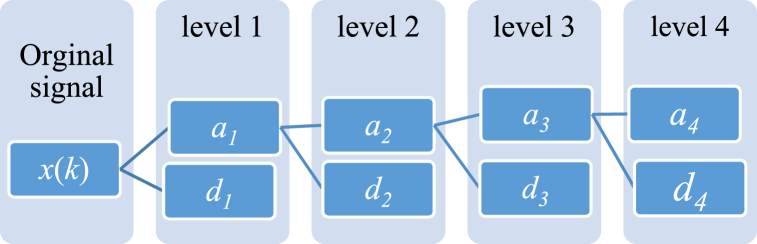
The structure is organized as in this level-4 decomposition diagram.

In the first and second levels of decomposition, the detailed components are denoted as d_1_ and d_2_, respectively. Correspondingly, the coarse components at these levels are represented by a_1_ and a_2_. Furthermore, at the *j*th level of decomposition, *c*_*j*_ signifies the coefficient pertaining to the coarse elements. Fig. 2 provides a schematic representation of a three-level DWT.

In DWT, the coarse segments associated with low frequencies are discernible at higher scales, while the fine segments related to high frequencies are observable at lower scales. These segments are linked to specific filters designed to suppress either high or low frequencies accordingly. Thus, for a thorough analysis of fault signal characteristics, it's crucial to establish parameters such as the Sampling frequency, the Wavelet basis type, and the Decomposition levels.

### 3.2 Classification using wavelet neural networks

Classification in data mining is employed to predict the category of each data instance. It involves developing a function that connects the input features to an output domain consisting of multiple classes. Neural Networks (NN) serve as a potent mechanism in pattern classification, utilizing both training and testing datasets to build a predictive model. ANNs have been adeptly applied through two principal strategies: firstly, by devising appropriate architectures and learning algorithms tailored for a variety of applications, and secondly, by amalgamating ANNs with other potent methodologies such as fuzzy logic, GA, DWT, or frequency transformations to enhance their performance. Owing to the importance of models and data originating from transformation functions within the frequency domain, Wavelets have become an instrumental resource when integrated with ANNs. The success of the networks is largely dependent on the caliber of both the training methodology and the data used for training. In this research, data preprocessing is conducted through the application of wavelet coefficients prior to training the neural network. Subsequently, a multi-layer backpropagation neural network is utilized for classification and fault detection. To validate the efficacy of the proposed WNN approach, test data is used to train a straightforward Multi-Layer Perceptron NN (MLPNN) as well as a WNN, with the outcomes of both compared thereafter.

#### 3.2.1 Methodology

**Fig. 3:**
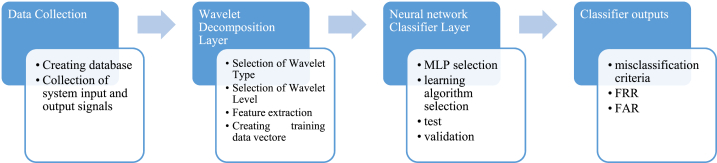
The block diagram of the classification algorithm.

The proposed WNN architecture comprises two distinct layers: the Wavelet layer and the Multi-Layer Perceptron layer, in that order. Fig. 3 illustrates the WNN classification algorithm as described.

#### 3.2.2 Wavelet layer

This layer is responsible for feature extraction from faulty signal. During the feature extraction phase, the DWT is applied to both the current and time-delayed input signal values (*x(k*) and *x(k-k*_*0*_*),* where *k0 = 1,2,6*), allowing them to be individually broken down into approximate and detailed features. The precise coefficients derived from these wavelets are subsequently forwarded to the neural network for training purposes. The significance of wavelet basis functions in classification performance is well-recognized. Consequently, throughout the development of this research methodology, a variety of wavelet functions—including Haar, Symlets, Daubechies, Coiflets, and Biorthogonal Wavelets—were evaluated at different levels. The selection of orthogonal functions was primarily due to their computational efficiency.

#### 3.2.3 Multi-layer perceptron (MLP)layer

**Fig. 4:**
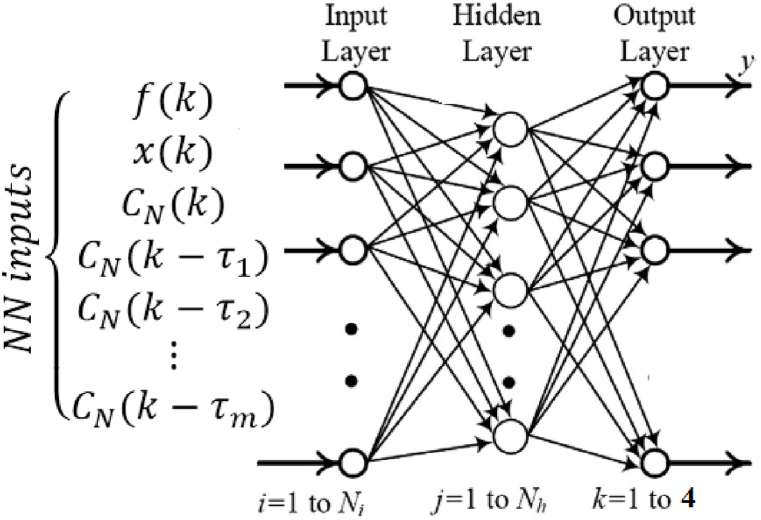
Structure of MLP classifier neural network for the fault location estimation. where $C_{N}\left(k\right)$ is vector of reconstructed approximate and detail coefficients, based on the wavelet decomposition structure of *x(k)*.

MLP stand out as a pivotal technique within the realm of machine learning, particularly under the ANN category. Diverging from the conventional linear classifiers, MLPs excel in capturing the complex patterns inherent in non-linear data. An MLP's architecture is a series of interconnected layers where each node, akin to a neuron, employs non-linear activation functions—except for the input layer nodes. The architecture also includes one or more hidden layers between the input and output layers, which are non-linear. To ensure the MLP neural network operates at its best, the training parameters and its structure were meticulously fine-tuned through extensive experimentation. This process involved varying the hidden layers' count and dimensions, adjusting learning rates, and testing different activation functions. The objective of this research was to investigate the efficacy of WT when applied as a preprocessing step for an ANN Faults Classifier within the Power Transformer Windings framework. Consequently, the specific training parameters and the architecture of the MLP employed are detailed in Table 1 and illustrated in Fig. 4. The input vector of presented MLP classifier neural network is concidered as Eq. (7):

(7) $$\text{Classifier}\;\text{NN}\;\text{input}={\left[\begin{array}{ccccccc} f\left(k\right) & x\left(k\right) & C_{N}\left(k\right) & C_{N}\left(k‐\tau_{1}\right) & C_{N}\left(k‐\tau_{2}\right) & \cdots & C_{N}\left(k‐\tau_{m}\right) \end{array}\right]}^{T}\text{,}$$

**Table 1:** ANN classifier outputs.

Transformer Output state	NN fault detector output		NN classifier outputs			
	Output 1	Output 2	Output 1	Output 2	Output 3	Output 4
Normal/Class I	1	0	1	0	0	0
axial displacement/Class II	0	1	0	1	0	0
radial deformation/Class III	0	1	0	0	1	0
short circuit turn/Class IV	0	1	0	0	0	1

In the proposed MLP classifier neural network, the input vector (Eq. (7)) is denoted as follows: f(k) represents the frequency and x(k) denotes the main signal. Furthermore, the term $C_{N}\left(k\right)={\left[\begin{array}{cccccc} a_{N}\left(k\right) & d_{N}\left(k\right) & d_{N‐1}\left(k\right) & \cdots & d_{2}\left(k\right) & d_{1}\left(k\right) \end{array}\right]}^{T}$ is defined as the vector of reconstructed coefficients at level N, where $a_{N}$ is the approximation coefficient and $d_{n=1\ldots N}$ are the detailed coefficients at level N, derived from the DWT. Additionally, $C_{N}\left(k‐\tau_{m}\right)$ is the delayed DWT signal coefficient with a pre-specified delay $\tau_{m}$. The output layer comprises four neurons, each representing a distinct fault condition: Healthy, AD, RD, and SC faults. The trained ANN in question is structured with three hidden layers, each containing 15, 30, and 8 neurons respectively, and utilizes a sigmoid transfer function. The output layer is composed of four neurons and employs a Soft Max transfer function, which is chosen over the sigmoid function for this layer. The classifier NN is trained using the Levenberg-Marquardt optimization algorithm, with the training process being halted upon reaching the minimum error. The network's training is capped at 500 epochs, and it allows for no more than 10 failed iterations.

Briefly, the process of selecting the most informative wavelet coefficients involved several key steps to ensure that the features used for fault detection and classification were both relevant and effective:

1. Wavelet Decomposition: The signal was decomposed using DWT into various levels of approximation and detail coefficients. In the first step according to the classification error rate Different wavelet types (e.g., Haar, Daubechies, Symlets, Coiflets) and decomposition levels were evaluated.
2. Feature Extraction: From the decomposed signal, both approximation and detail coefficients were extracted. These coefficients represent different frequency components of the signal, capturing both high-frequency and low-frequency information.
3. Logistic Regression with LMG Metric: Logistic regression, augmented by the LMG (Lindeman, Merenda, Gold) metric, was employed to assess the relative importance of each wavelet coefficient. The LMG metric helps in quantifying the contribution of each coefficient to the classification accuracy.

## 4 Data analysis

To analyze and construct models that show how several quantitative variables $X_{1},X_{2},\ldots ,X_{k}$ influence a quantitative outcome Y, we typically use multiple linear regression. However, when the goal is to understand the connection between one or more quantitative variables $X_{1},X_{2},\ldots ,X_{k}$ and a qualitative outcome Y, linear regression falls short. In such scenarios, logistic regression is the appropriate method. For a binary outcome, binary logistic regression is used, while for outcomes with more than two levels, ordinal logistic regression is applied. Essentially, logistic regression is a classification method that also quantifies the impact of each predictor variable. In practice, the significance of $X_{1},X_{2},\ldots ,X_{k}$ is often assessed through standardized coefficients, which can present certain limitations and challenges.

Data were sequentially inputted into the model according to their volume and processed with R software, version 4.3.0. The impact of neural network (NN) training variables x(k), f(k), and the reconstructed coefficient vector $C_{N}\left(k\right)={\left[\begin{array}{cccccc} a_{N}\left(k\right) & d_{N}\left(k\right) & d_{N‐1}\left(k\right) & \cdots & d_{2}\left(k\right) & d_{1}\left(k\right) \end{array}\right]}^{T}$ on classification was assessed using multiple logistic regressions, guided by their relative significance. This regression method accounts for the linear effects of NN training factors f(k) and the coefficient vector C N (k). Due to interrelations among NN training variables, some effects may be negligible (p > 0.05). Stepwise selection is known for its pitfalls, including artificially low p-values that don't account for multiple fittings and skewed R^2^ values [41]. To pinpoint the optimal model, standard metrics like the Bayesian information criterion, Akaike information criterion, and adjusted R^2^ are employed. Various approaches for variable selection have been proposed, such as in Ref. [42]. A typical technique to assess the importance of predictor variables is through standardized regression coefficients. Nonetheless, this method encounters various issues as outlined in Refs. [43,44]:

• When there is multi-co-linearity, regression coefficients such as standard regression coefficients cannot be understood.
• Multi-co-linearity can cause not only large distortions in the estimates of the sizes of the regression coefficients but also changes in their directions.
• When there is multi-co-linearity, regression coefficients cannot be trusted as measures of relative significance, because they do not split R^2^ naturally.

**Fig. 5:**
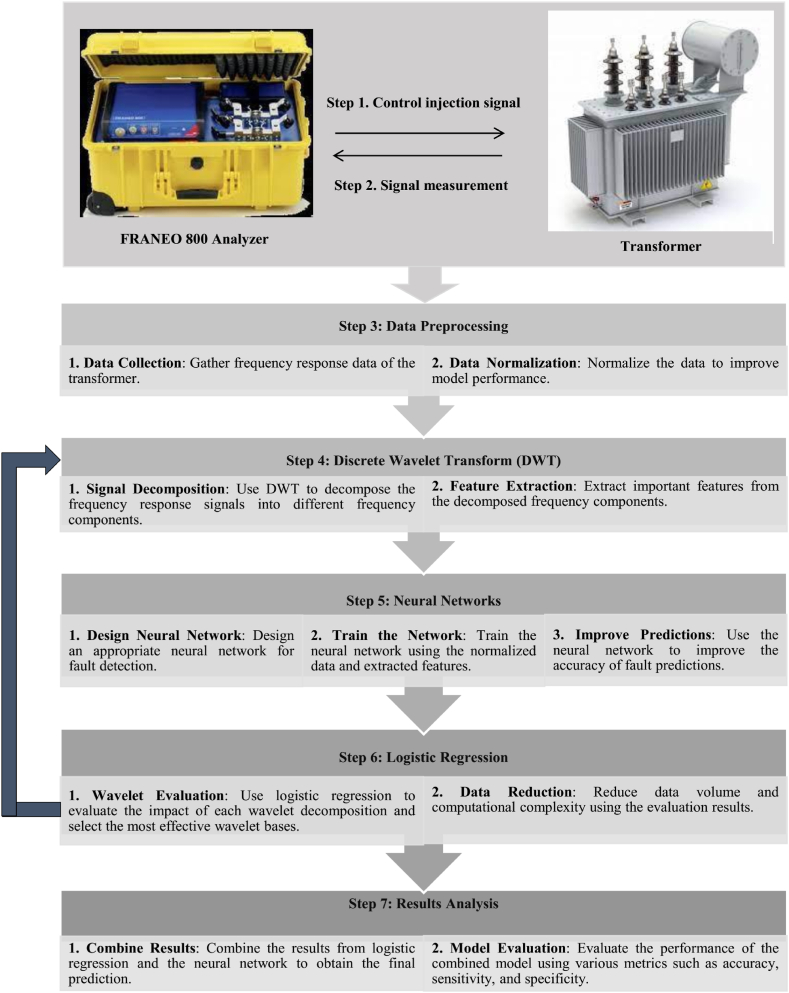
Steps to implement the proposed method for transformer fault diagnosis.

Several strategies exist for partitioning R^2^ to tackle this problem. The LMG technique, developed by Lindeman, Merenda, and Gold, is one such measure and is available through the R package “relaimpo” [45]. Additionally, multiple methods are evaluated against each other to select variables [46,47,48]. In this study, logistic regression was employed alongside the LMG measure of relative importance to analyze the data gathered. Fig. 5 highlights the detailed methodology employed in this simulation process.

These steps are summarized as follows:

Step 1 *Frequency Response Measurement*: In this stage, frequency response measurements are conducted on transformers in both healthy and faulty states using the RANEO 800 analyzer. The goal is to compile a comprehensive database of frequency responses for healthy and faulty transformers, which will serve as the basis for subsequent analyses.
Step 2 *Data Collection*: The frequency response data obtained in the previous stage are calculated and analyzed. This analysis involves examining various characteristics of the frequency response for healthy and damaged windings. The aim is to identify and extract key features from the frequency response data that can aid in fault detection.
Step 3 *Feature Extraction*: In this stage, DWT is applied to the frequency response data to extract relevant features.
Step 4 *Neural Network Training*: The features extracted in the previous stage are used as inputs to an ANN. This network is trained using both healthy and faulty data. The objective is to develop a diagnostic model capable of accurately identifying and classifying transformer faults. Logistic Regression is then used to assess the impact of each wavelet decomposition. The goal is to identify the most effective wavelet bases and reduce the data volume to improve computational efficiency and fault detection accuracy.
Step 5 *Validation and Testing*: The performance of the ANN is evaluated using confusion matrices and other statistical measures. This evaluation includes assessing accuracy, sensitivity, specificity, and other performance metrics. The goal is to ensure the diagnostic model's accuracy and reliability in identifying and classifying transformer faults.

## 5 Case study

The tested case is a 1.2 MVA, 20/0.4 kV, 3phase, 50 Hz, D/Yg transformer with the HV and LV windings made up of two continuous layers and 45 interleaved disks, with 9 turns in each layer and 20 turns in each disc, respectively. Moreover, the HV/LV windings' height, core length/height, LV and HV windings' outer/inner diameter are 834/893, 3652/976, 763/741, 916/846 mm, respectively. The arrangement's internal nodes have to be accessed by us. Kraft paper and mineral oil make up the transformer's insulation. To obtain the transformer transfer function, researchers artificially simulated ten distinct level of three types of the AD, RD, and SC faults in each of the three-phase windings of the transformer (A, B, and C, respectively) at different degrees and locations.

In the present research, axial displacements are implemented by shifting the HV winding in relation to the LV winding in 5 mm steps, up to a total of 50 mm (which equates to 6 % of the winding's height) to obtain different degrees of this fault. In the simulated RD fault, faults are introduced in multiple directions (one, two opposing, three, and four directions); however, the ratio of radial deformation to the average radius of the disk is consistently maintained at 2 %, 4 %, and 6 %. To simulate SC faults, different parts of the HV winding can be short-circuited. For implementation of this work and better access to the winding, connectors have been connected in different places of winding so that different number of turns can be short-circuited to create SC fault and simulate different degrees of fault. The SC fault level is determined by the ratio of short-circuited turns to the total turns in the winding.

The study employs an Omicron FRANEO 800 sweep FRA analyzer to capture transfer function responses. This device operates according to the IEC 60076-18 standard, which is an internationally recognized standard for testing and evaluating transformers. The data collection process with this device involves several key steps. First, the device is connected to the transformer, and initial settings are configured. Then, the device applies a frequency signal in the range of 100 Hz to 1 MHz to the transformer and measures its frequency response. The collected data in each simulation includes 1622 sample points uniformly distributed across the frequency range. These data are digitally stored and then analyzed using the proposed methods to identify any mechanical or electrical changes in the transformer. During the FRA measurements, the ambient temperature was 27 °C and the relative humidity and atmospheric pressure was 40 percent and 1015 hPa, respectively.

The simulations were conducted using MATLAB software. Classification tools are employed to process various derived features as inputs and categorize the type of anomaly detected as outputs, which can be classified as either an electrical fault (SC) or mechanical faults (RD and AD). During the simulation phase, for each type of disturbance, feature sets numbering 8 for SC faults, and 6 each for RD and AD faults, are utilized based on the signals acquired. This collection of data is subsequently referred to as the main database within the paper. To demonstrate the efficacy of the proposed method, 35 % of this dataset is allocated for training—comprising 4 sets each of AD and RD defects, along with 6 sets of SC faults—while the remainder is designated for testing purposes. Despite the fact that a mere 35 % of the dataset is employed in the development of the WNN, the outcomes demonstrate the high precision of the method across all samples in the database. The objective of the proposed approach is to accurately detect both AD and RD defects, as well as electrical fault (SC).

### 5.1 Simulation results

**Fig. 6:**
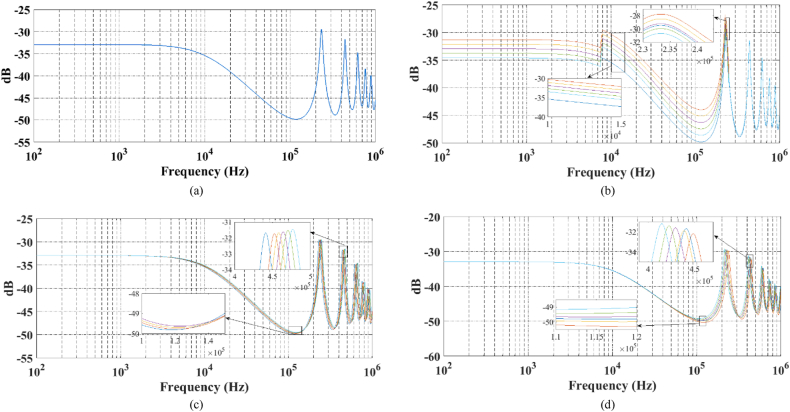
a) Healthy, b) Short Circuit, c) Axial Displacement, d) Radial Deformation.

The TF is calculated across a wide frequency spectrum, reaching up to 1 MHz. Fig. 6a–d display the frequency response characteristics for both intact and damaged windings, including RD, AD, and SC defects, respectively.

As previously noted, the analysis of FRA outcomes (in the figures above) is a complex field that necessitates the expertise of specialists to identify the nature of faults. Consequently, the transfer functions derived from these measurements serve as input data for the DWT and ANN for fault detection purposes.

## 6 Results and discussion

To demonstrate the effectiveness of the proposed method, approximately 26,000 samples comprising both healthy and defective data, representing various degrees of faults in the transformer, were chosen across 2000 frequencies ranging from 1 Hz to 1 MHz. This dataset is referred to as the ‘main database’ in the subsequent sections of the paper. In the proposed framework, two distinct ANN architectures have been formulated. The initial architecture is designed for fault detection, featuring two outputs, while the second, termed as the classifier, encompasses four outputs. A variety of configurations for both the detection and classification neural network architectures—including the count of neurons and hidden layers—were systematically assessed. The optimal architecture for the neural network's hidden layers was determined to be a 15-30-8 structure.

### 6.1 Fault detection (wavelet-based fault detector)

**Fig. 7:**
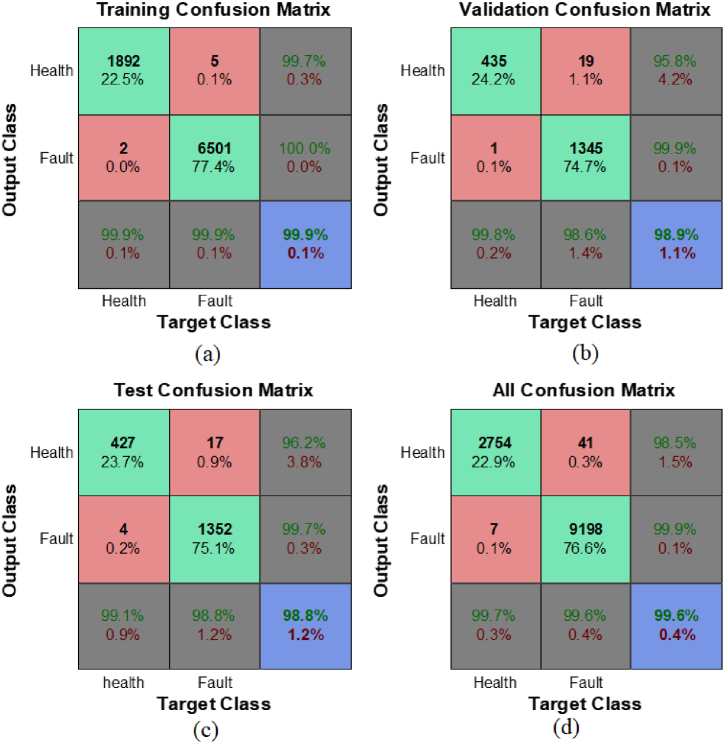
(a) Train data, (b) Validation data, (c) Test data and (d) Total data Confusion matrices of NN fault detector with Db4-level DWT.

In the first phase, a wavelet-based neural network approach is utilized to distinguish internal faults from the standard operational conditions of the power transformer. The input layer's size is dictated by the chosen level of DWT and the number of delays ($\tau_{1}=1,\tau_{2}=2,\tau_{3}=6$), while the output layer consists of merely two neurons. The ANN's target output corresponds to the values listed in Table 1. For the training and evaluation of the neural network, a total of 12,000 data samples were randomly extracted from the main database. Out of these, 70 % were allocated for training, and the remaining 30 % were used for testing and validation. In order to show the effect of the use of DWT on improving the performance of the neural network in fault detection (without determining the type of fault), Table 2 shows the comparison of the performance of the wavelet Haar and Db4 and the classical ANN. It should be mentioned that when using DWT, the number of NN layers is considered unchanged. Also, the detail of confusion matrices for the neural network classifier, which employs Daubechies wavelet (db4 level 3) preprocessing, are depicted in Fig. 7.

**Table 2:** ANN and ANN-DWT Classifier comparison.

	Accuracy (%)	Sensitivity (%)	Specificity (%)	CPU Time (300 iteration)
ANN	83.59	4.9	89.33	138s
NN + DWT(Haar-level3)	95.36	86.6	95.0	376s
NN + DWT(Db4-level3)	99.6	99.75	99.6	375s

It can be seen that the sensitivity rate in the classical NN is extremely low, which shows the very weak ability of the classical NN in detecting this type of faults. The simulation data reveals that the Db4 wavelet-based detector has the lowest average misclassification rate at just 0.4 %. For the test and validation datasets, the misclassification rate remains below 1.2 %, as shown in Fig. 7. Additionally, Fig. 7 indicates that the Db4 wavelet yields a high success rate of 99.6 %, coupled with a low false rejection rate (FRR) of 0.1 % and a false acceptance rate (FAR) of 0.4 %. This implies that in 0.1 % of cases, healthy data is erroneously identified as faulty, and in 0.4 % of cases, fault data is mistakenly recognized as normal.

### 6.2 Fault classifier

In the subsequent stage, both the MLP NN and the wavelet-based neural network classifiers are employed to analyze the distinctions between various fault systems and the healthy system.

#### 6.2.1 MLP NN classifier

In the following, the paper proceeds to detail the NN classifier's efficacy in discerning various levels of short circuits, radial deformations, and axial displacement defects. Furthermore, to validate the reliability of these findings and to showcase the model's accuracy in detecting mechanical and electrical anomalies, the transformer is subjected to an array of RD, AD, and SC fault conditions for assessment. For the training and evaluation of the classifier neural network, a total of 9000 data samples were randomly chosen from the main database to form a new dataset, referred to as the ‘training database’. Within this training database, 1000 samples were selected from healthy data across different frequencies, and 8000 samples were from faulty data. Of the entire 9000 samples in the training database, 70 % were used to train the classifiers, while the remaining 30 % were divided equally for validation and testing purposes, with 15 % for each. To demonstrate the efficacy of the proposed method, for the purpose of assessing the classification algorithm, a set of 17,000 new test samples—representing various fault levels of the transformer—was selected. These samples were then applied to the previously trained neural network, from which the classification outcomes were derived. The simulation results based on these outcomes will be presented accordingly.

**Fig. 8:**
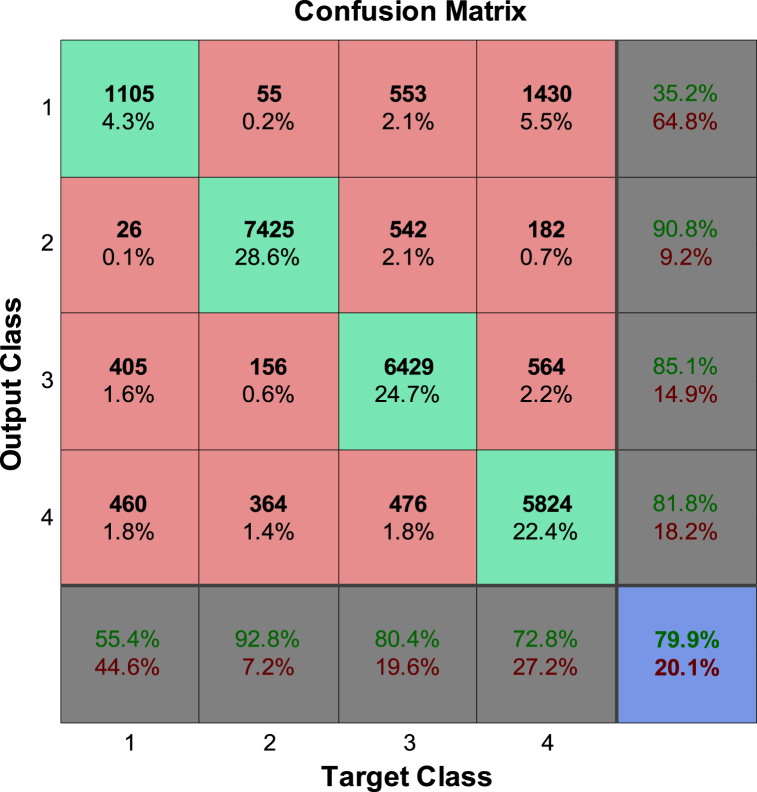
Confusion matrices of trained NN classifier without DWT preprocessing for main database.

The performance analysis is conducted by assessing the generalization capability, classification accuracy, and confusion matrix against a foundational dataset comprising 26,000 data samples. In the initial phase of the feature extraction process, both current and delayed input signal values (*x*(k) and *x*(k-k_0_)), where (k_0_ = 1,2,6) are submitted to the neural network for training. With the aim of achieving the best neural network performance in fault classification, an efficient neural network structure including an optimal number of middle layer neurons has been selected with the help of genetic algorithm. It is clear that the selection of non-optimal structures for the desired neural network weakens the performance of the classifier. The confusion matrices for the MLP NN classifier, which does not utilize DWT preprocessing, are illustrated in Fig. 8.

The simulation results indicate that the misclassification rate for the NN classifier without DWT preprocessing exceeds 20 % (79.9 % accuracy rate), which is not considered satisfactory. Additionally, there is a high percentage of both FRR and FAR. While the outcomes for radial deformation faults may be somewhat acceptable, the FRR and FAR for the healthy class are deemed unacceptable.

#### 6.2.2 Wavelet-based neural network

In the next step, utilizing DWT, the NN is trained using the detailed coefficients of the wavelets. Table 3 presents the outcomes for a selection of widely-used Mother wavelets. The classification results, specifically the Correct Classification Rate, were computed for the main testing dataset, which includes all 26,000 samples.

**Table 3:** The results of the proposed method computed over several DWT levels (Overall Accuracy Rate%).

Harr(‘haar’)		Level 1	Level 2	Level 3	Level 4	Level 5	Level 6	Level 7
		95.82	90.44	79.46	83.48	78.09	76.92	95.26
Daubec hies wavelet (‘db’)	db2	96.55	97.3	95.63	94.9	93.77	89.47	60.52
	db3	95.13	96.75	97.01	85.75	88.60	71.28	67.47
	db4	95.43	94.79	67.65	95.21	83.04	78.51	66.02
Symlets (‘sym’)	Sym1	95.73	90.443	79.5	83.9	78.09	76.92	89.26
	Sym2	96.11	96.55	95.2	97.17	93.7	89.1	61.02
	Sym4	72.87	93.51	97.07	97.10	90.58	89.38	69.60
	Sym5	94.12	96.34	96.72	95.01	94.52	77.81	75.17
Coiflets	Coif1	96.32	97.50	96.40	95.67	93.618	79.70	67.01
	Coif2	96.72	96.16	95.26	94.81	91.57	78.79	67.25
Biorthogonal	Bior1.1	92.89	90.1	79.8	83.50	77.1	76.8	90.16
	Bior2.6	97.01	95.23	95.13	94.98	88.96	83.86	70.44
Reverse Biorthogonal		95.81	90.44	79.46	83.48	78.08	76.92	95.26

The most favorable results were achieved using Symlets and Coiflets as Mother wavelets, which yielded the highest rates of correct classifications and the lowest instances of misclassified data samples. Additionally, db3 and Bior2.6 wavelets also produced reasonably good outcomes. These classifiers demonstrated robust performance across both the training and the main datasets. Moreover, they exhibited a considerable degree of resilience against typical faults, as evidenced by the minimal count of misclassifications. It can also be seen from Table 3 that the use of higher estimation levels generally does not improve the performance of the neural network, but the performance of the network is weakened due to the addition of less important details (such as noise) to the training data of the network. In order to more closely examine the performance of different types of wavelet transforms simulated in Table 3 in comparison with each other and common MLP neural networks, more detailed parameters such as sensitivity, selectivity and accuracy separately for a number of wavelet bases that have relatively higher accuracy. and misclassification index are presented in Table 4.

**Table 4:** Result of selected DWT-NN classifiers including accuracy, sensitivity and specificity (%).

		Axial displacement fault (%)			Radial deformation fault (%)			Short circuit turn fault (%)			CPU Time (300 Iteration)
		Sensitivity	Specificity	Accuracy	Sensitivity	Specificity	Accuracy	Sensitivity	Specificity	Accuracy	
**ANN**		88.9	90.68	90	80.3	92.7	88.5	72.8	92	85.6	345s
**ANN + DWT**	Db2-level2	99.4	99.05	99.12	96.64	99.12	98.35	98.05	98.9	98.6	500s
	Sym4-level3	98.05	98.93	98.62	97.5	98.95	98.5	96.76	98.91	98.24	562s
	Coif1-Level2	98.64	99.18	99.01	97.45	99.17	98.64	97.11	98.95	98.38	533s
	Bior2.6	98.3	99.3	99	96.21	99.28	98.32	96.9	98.6	98.1	400s

**Fig. 9:**
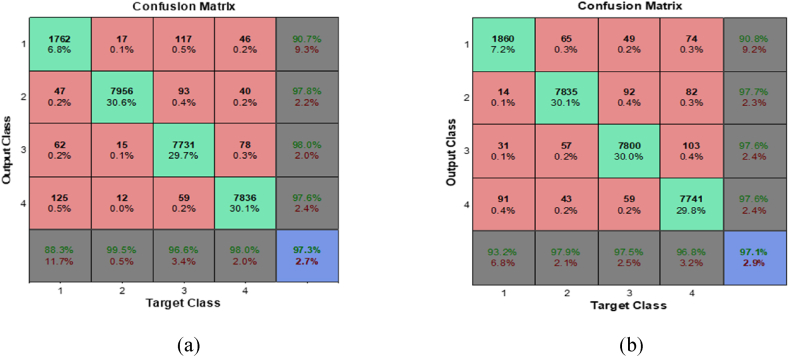
Confusion matrices of NN classifier with DWT preprocessing for (a) ‘Db2-D2’ and (b) ‘sym4 – level3’ mother wavelet for main database.

The results show that the neural network obviously provides weaker results in fault detection. Also Confusion matrices for the classifiers, which incorporate the Db2 – D2 and sym4 – level3 wavelet, are provided in Fig. 9(a). The simulation data indicates that the misclassification rate for the comprehensive main database—which includes the training database plus an additional 17,000 new test samples, totaling 26,000 data points—is 2.7 %, as depicted in Fig. 9. Notably, Class 1 (the Healthy class) exhibits the highest False Acceptance Rate (FAR) and False Rejection Rate (FRR), contributing to the overall misclassification rate.

**Fig. 10:**
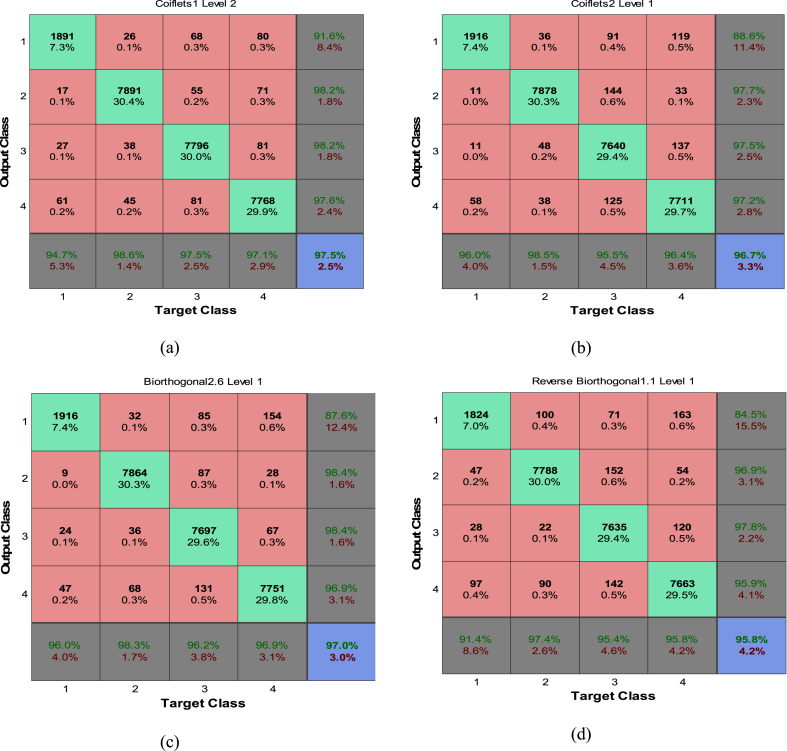
Confusion matrices of NN classifier with (a) Coiflets1-level2, (b) Coiflets2-level1, (c) Biorthogonal-level1 and (d) Reverse biorthogonal-level1.

Fig. 9b illustrates that the WNN classifier, when utilizing the Sym4-Level3 wavelet, achieves a reduction in misclassification to 2.9 %. Additionally, it is observed that the misclassification metrics—FRR and FAR—show enhancement relative to the Db2-D2 wavelet. For the sake of comparison, confusion matrices for various WNN classifiers that exhibit low misclassification rates are exhibited in Fig. 10.

### 6.3 Effective parameters on classification

To evaluate the impact of both approximation and detail coefficients from wavelet analysis on minimizing classification errors, data from a three-level analysis using db2, Sim4, and Sim5 wavelets were examined. The dataset in question was subjected to logistic regression analysis, which utilizes the LMG metric to determine the relative importance of variables. The influence of various *f*(k) factors, along with the approximation and detail coefficients, on the classification process has been thoroughly examined and the findings are consolidated in Table 5, Table 6.

**Table 5:** Output of the logistic regression technique based on LMG relative importance metric for db2, Sim4 and Sim5, 3-level wavelet decomposition.

Parameter	sim5 level3					db2 level3					sim4 level3				
	Fault			LMG (%)	10-fold CV Accuracy	Fault			LMG (%)	10-fold CV Accuracy	Fault			LMG (%)	10-fold CV Accuracy
	AD	RD	SC			AD	RD	SC			AD	RD	SC		
*F*	ns	ns	Ns	0.0	98.5	ns	ns	ns	0.0	97.8	ns	ns	ns	0.0	98.1
*a*_*1*_	–	+	+	19.6		+	–	+	18.5		–	+	+	19.9	
*d*_*1*_	+	+	–	33.5		+	+	–	35.6		+	+	–	35.1	
*a*_*2*_	ns	ns	ns	1.8		ns	ns	ns	1.9		ns	Ns	ns	1.9	
*d*_*2*_	+	–	–	18.1		–	+	–	16.6		+	–	–	20.6	
*a*_*3*_	+	+	–	12.8		–	+	–	12.8		–	+	+	11.6	
*d*_*3*_	+	+	–	14.2		–	+	–	14.6		–	+	+	10.9	

ns: no significant.

**Table 6:** Output of the logistic regression technique based on LMG relative importance metric for 4-level wavelet decomposition db2.

Parameter	A	R	SC	LMG (%)	10-fold CV Accuracy
*F*	ns	ns	ns	0.0	97.7
*a*_*1*_	+	–	+	18.2	
*d*_*1*_	+	+	–	29.6	
*a*_*2*_	ns	ns	ns	4.4	
*d*_*2*_	–	+	–	13.8	
*a*_*3*_	ns	ns	ns	3.5	
*d*_*3*_	ns	ns	ns	7.9	
*a*_*4*_	–	–	+	13.1	
*d*_*4*_	ns	ns	ns	9.6	

ns: no significant.

Table 5 indicates that for sim5 level3, the set $\left\{f,a_{2}\right\}$ had no significant impacts. For axial displacement fault, the set $\left\{d_{1},d_{2},a_{3},d_{3}\right\}$ had positive impact and the set $\left\{\;a_{1}\right\}$ had negative impact. Moreover, for radial deformation fault, the set $\left\{a_{1},d_{1},a_{3},d_{3}\right\}$ had positive impact and the set $\left\{\;d_{2}\right\}$ had negative impact. Furthermore, for short circuit turn fault, the set $\left\{\;a_{1}\right\}$ had positive impact and the set $\left\{d_{1},d_{2},a_{3},d_{3}\right\}$ had negative impact. For db2 level3, the set $\left\{f,a_{2}\right\}$ had no significant impacts. For axial displacement fault, the set $\left\{{\;a_{1},d}_{1}\right\}$ had positive impact and the set $\left\{d_{2},a_{3},d_{3}\right\}$ had negative impact. Moreover, for radial deformation fault, the set $\left\{d_{1},d_{2},a_{3},d_{3}\right\}$ had positive impact and the set $\left\{a_{1}\right\}$ had negative impact. Furthermore, for short circuit turn fault, the set $\left\{d_{1},d_{2}\right\}$ had positive impact and the set $\left\{a_{1},a_{3},d_{3}\right\}$ had negative impact. For sim4 level3, the set $\left\{f,a_{2}\right\}$ had no significant impacts. For axial displacement fault, the set $\left\{d_{1},d_{2}\right\}$ had positive impact and the set $\left\{{a_{1},a}_{3},d_{3}\right\}$ had negative impact. Moreover, for radial deformation fault, the set $\left\{a_{1},d_{1},a_{3},d_{3}\right\}$ had positive impact and the set $\left\{d_{2}\right\}$ had negative impact. Furthermore, for short circuit turn fault, the set $\left\{{a_{1},a}_{3},d_{3}\right\}$ had positive impact and the set $\left\{d_{1},d_{2}\right\}$ had negative impact.

The parameter d_1_ emerged as the most significant, boasting the highest LMG value as indicated in Table 5. It is noteworthy that the frequency component's contribution to diminishing classification error is registered as zero. This is not indicative of the frequency's insignificance in data analysis, but rather a reflection of its influence being encapsulated within other data derived from the wavelet transformation. To reduce over-fitting, 10-fold cross-validation (CV) technique was used. This technique verified the reliability of logistic regression.

Table 6 indicates that for db2level4, the set $\left\{f,a_{2},a_{3},d_{3},d_{4}\right\}$ had no significant impacts. For axial displacement fault, the set $\left\{{a_{1},d}_{1}\right\}$ had positive impact and the set $\left\{d_{2},a_{4}\right\}$ had negative impact. Moreover, for radial deformation fault, the set $\left\{\;d_{1},d_{2}\right\}$ had positive impact and the set $\left\{\;a_{1},a_{4}\right\}$ had negative impact. Furthermore, for short circuit turn fault, the set $\left\{\;a_{1},a_{4}\right\}$ had positive impact and the set $\left\{\;d_{1},d_{2}\right\}$ had negative impact. The parameter d_1_ emerged as the most significant, boasting the highest LMG value. CV technique verified the reliability of logistic regression.

## 7 Conclusion

This research introduced an innovative method for detecting transformer winding faults by integrating logistic regression, DWT, and ANN. The use of logistic regression to evaluate the impact of wavelet decompositions and select the best wavelet bases as inputs to the ANN is the main innovation of this research, which has been less addressed in previous studies. This method showed significant improvements in accuracy and efficiency compared to previous methods that used only ANN, DWT, or a combination of both. The results indicate that the proposed method enhances the accuracy and efficiency of fault detection systems and reduces maintenance costs. Additionally, the results reveal that omitting DWT preprocessing significantly increases the conventional NN classifier's error rate to over 20 %, yielding unsatisfactory results. High FRR and FAR percentages are also observed, and while the detection of axial displacement and radial deformation faults is somewhat satisfactory, the accuracy for identifying healthy conditions is not. However, the simulations demonstrate a remarkable success rate for the proposed WNN in fault classification, with an accuracy rate nearing 98 % for pinpointing electrical fault at the HV terminal of the transformer, resulting in acceptable FRR and FAR figures. Notably, the simulations underscore that employing the ‘Db2-D2’ mother wavelet within the WNN classifier significantly enhances the detection of electrical SC faults, achieving nearly a 98 % success rate and reducing the misclassification rate to just 2 %. These findings suggest that the proposed method can serve as a strong foundation for future research in transformer fault detection and offer a promising approach to enhancing the reliability and efficiency of power systems.

For future research, the following suggestions are provided:

1. *Development of online monitoring systems*: Design and implementation of online monitoring systems for early fault detection and reduction of transformer downtime.
2. *Improvement of machine learning algorithms*: Optimization of machine learning algorithms such as deep neural networks (DNN) and convolutional neural networks (CNN) to increase accuracy and reduce computational complexity.
3. *Utilization of big data*: Use of data mining techniques and big data analysis to identify hidden patterns and improve fault detection accuracy.
4. Testing on Larger Datasets and More Real-World Cases: It is proposed that in future work, the proposed method be tested on larger datasets and more real-world cases. This would help to further validate the findings and ensure the robustness of the proposed method in diverse and practical settings.

## CRediT authorship contribution statement

**Salman Baroumand:** Conceptualization, Data curation, Formal analysis, Methodology, Resources, Software, Validation, Writing – review & editing. **Ali Reza Abbasi:** Writing – review & editing, Writing – original draft, Validation, Supervision, Software, Resources, Project administration, Methodology, Investigation, Formal analysis, Data curation, Conceptualization. **Mohammadreza Mahmoudi:** Writing – review & editing, Writing – original draft, Visualization, Validation, Software, Resources, Methodology, Investigation, Data curation, Conceptualization.

## Data availability

The data and code can be requested from corresponding author.

## Declaration of competing interest

The authors declare that they have no known competing financial interests or personal relationships that could have appeared to influence the work reported in this paper.
